# Supplementary material for: A Tetra-Panel of Serum Circulating miRNAs for the Diagnosis of the Four Most Prevalent Tumor Types
Source: Int J Mol Sci. 2020 Apr 16;21(8):2783. doi: 10.3390/ijms21082783 (PMC7215589; doi:10.3390/ijms21082783)
Supplement: Supplementary file 1 [file ijms-21-02783-s001.pdf]

Supplementary material

Table S1. List of PCR assays for specific miRNAs according to tumor type.

**Tumor type**	**miRNA**	**Assay name/ Assay Id**
**BC**	miR-21	hsa-miR-21. Id: 000397
	miR-155	hsa-miR-155. Id: 002623
	miR-205	hsa-miR-205. Id: 000509
**LC**	miR-21	hsa-miR-21. Id: 000397
	miR-429	hsa-miR-429. Id: 001024
	miR-205	hsa-miR-205. Id: 000509
	miR-200b	hsa-miR-200b. Id: 001800
	miR-203	hsa-miR-203. Id: 000507
	miR-125b	hsa-miR-125b. Id: 000449
	miR-34b	hsa-miR-34b. Id: 002102
**CRC**	miR-21	hsa-miR-21. Id: 000397
	miR-92a	hsa-miR-92a. Id: 000431
	miR-29a	hsa-miR-29a. Id: 002112
	miR-210	hsa-miR-210. Id: 000512
	miR-221	hsa-miR-221. Id: 000524
	miR-200c	hsa-miR-200c. Id: 002300
	miR-155	hsa-miR-155. Id: 002623
**PCa**	miR-21	hsa-miR-21. Id: 000397
	miR-141	hsa-miR-141. Id: 000463
	miR-375	hsa-miR-375. Id: 000564
	miR-125b	hsa-miR-125b. Id: 000449
	miR-222	hsa-miR-222. Id: 002276
	miR-182	hsa-miR-182. Id: 002334
**Housekeeping**	miR-16	hsa-miR-16. Id: 00391
**Housekeeping**	U6snRNA	U6 snRNA. Id: 001973
**Housekeeping**	miR-1228	Hsa-miR-1228. Id: 002919

Hsa (Homo sapiens)

Table S2. **Summary of the selected miRNAs for each tumor type**

Cancer type	miRNA	Biological miRNA function	Observations	References
BC	miR-21	Targets and inhibits tropomyosin 1 (alpha) (TPM1), programmed cell death 4 (neoplastic transformation inhibitor) (PDCD4), and phosphatase and tensin homolog (PTEN) and other tumor-related genes.	Expression associated with tumor progression and poor prognosis.	[1–5]
	miR-155	Promotes mammary gland epithelial cell migration and invasion by targeting the tumor suppressors TP53INP1 and RhoA, respectively. In addition, induces B-cell malignancies by targeting Ship and C/EBPβ.	Overexpressed in cancer patients.	[6–8]
	miR-205	Down-regulates ErbB2, and meanwhile the expressions of cyclin E, cyclin D1 and cyclin-dependent kinase 6 (CDK6) are upregulated.	Overexpressed in cancer patients.	[9,10]
LC	miR-21	Negatively modulating the expression of tumor-suppressor genes.	Defined as a good diagnostic biomarker.	[11]
	miR-429; miR-205; miR-200b; miR-203 miR-125b; miR-34b	MiR-200 family and miR- 205 play critical roles in regulating EMT by targeting the E-cadherin repressors ZEB1 and ZEB2. Furthermore, ZEB1 has been reported to repress miR-203 in the ZEB/ miR-200 feedback loop. MiR-125b can directly target the tumor suppressor gene TP53 and the downstream modulator TP53INP1, contributing to malignancy. Interestingly, miR-34b, which is a transcriptional target of TP53 often found downregulated in tumor. miR-429 regulates E-cadherin transcriptional suppressors (ZEB1 y ZEB2)	This panel of genes was defined in collaboration with the Departments of Genetics and Oncology of the Institute for Cancer Research, OUS Radiumhospital et (Oslo, Norway) in the context of the EurocanPlatform project [FP7-NoE-EurocanPlatform- GA no.: 260791].	[12]
PCa	miR-21	Negatively modulating the expression of tumor-suppressor genes.	Overexpressed in cancer patients and	[13]

			predicts hormone resistance.	
	miR-141; miR-375	These miRNAs are epithelial specific, highly related in sequence and have known roles in maintaining the epithelial state by suppression of the epithelial-to mesenchymal transition.	Associated with tumor progression.	[14]
	miR-125b; miR-222; miR-182	MiR-125b has been reported to target BAK1 (a pro-apoptotic member of the BCL-2 gene family) and EIF4EBP1 (Eukaryotic translation initiation factor 4E-binding protein 1, a gene that encodes one member of a family of translation repressors proteins) and the transcriptional co-repressors NCOR2/SMRT. MiR-222 is involved in ADT-RCaP (androgen deprivation therapy recurrent CaP). MiR-182 downregulates MeCP2, which regulates CREB1 and MEF2C.	Overexpressed in cancer patients and provide prognostic information.	[15,16]
CRC	miR-21	MiR-21 downregulates tumor suppressor genes expression.	Overexpressed in cancer patients.	[17]
	miR-92a	MiR-92a induces EMT and regulated cell growth, migration and invasion, via suppression of PTEN expression.	Upregulated in cancer patients and promotes epithelial mesenchymal transition.	[18,19]
	miR-29a	MiR-29a regulates MMP2/E-cadherin through direct targeting KLF4.	Promotes CRC and its metastasis by regulating matrix metalloproteinase and E-cadherin.	[19,20]
	miR-210	MiR-210 modulates endothelial cell response to hypoxia and inhibits the receptor tyrosine kinase ligand Ephrin-A3.	Hypoxia-induced miRNA whose overexpression can stimulate angiogenesis	[21]

			and migration of epithelial cells.	
	miR-221	MiR-221 targets CD117, which then prevents cell migration and proliferation in endothelial cells. MiR-221 is known as an anti-angiogenic miRNA.	Its expression is associated with progression parameters such as dedifferentiation and p53 expression.	[22]
	miR-200c	Crucial inhibitor of epithelial-to-mesenchymal transition (EMT). Negative regulates its gene targets (ZEB1, ETS1 and FLT1), which in turn regulate E-cadherin and vimentin expression to trigger an EMT switch.	Up-regulated in CRC liver metastases, playing an important role in mediating metastatic behaviour.	[23]
	miR-155	Regulates the tumor suppressors TP53INP1 and RhoA.	Overexpressed and involved in tumor progression.	[24]

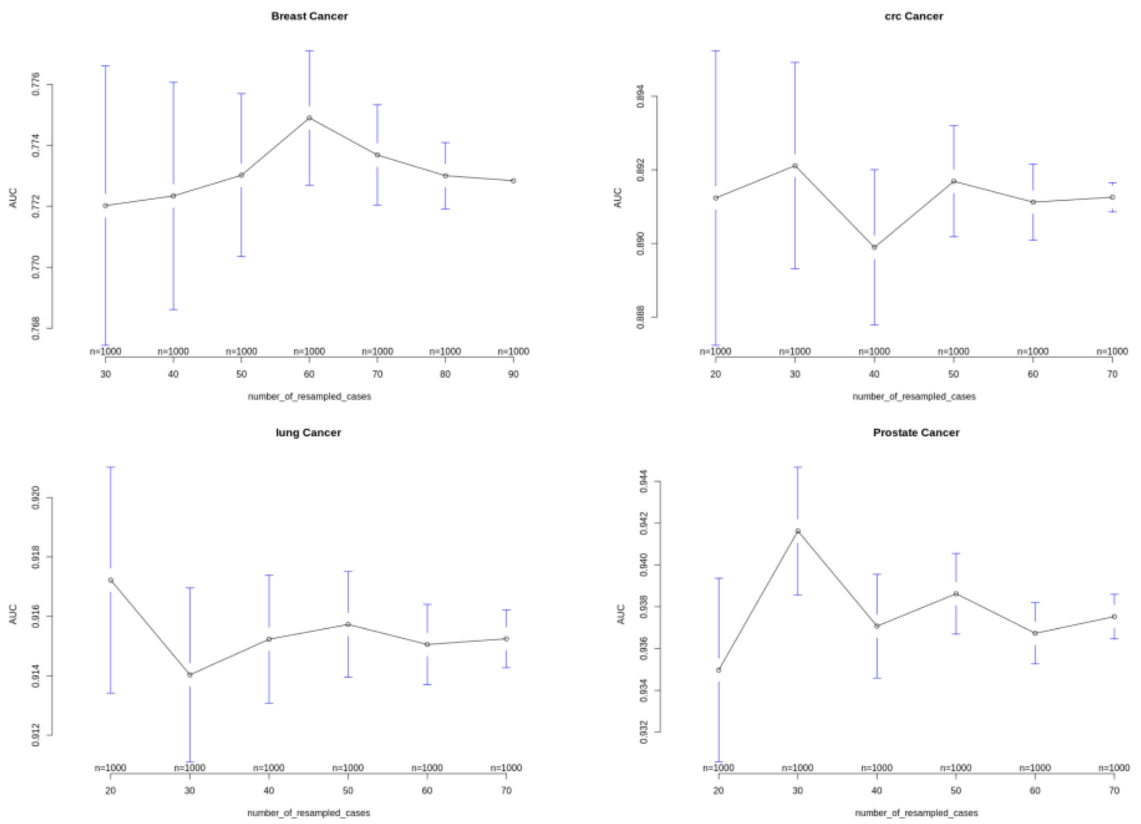

Figure S1. Cross validation of the AUC with 1000 interactions at different sample sizes for each tumor type. The AUC values trend to stabilize its value with the growing number of samples to the value estimated in our work. These interactions have been performed taking samples at random and reanalyzing the ROC curves. Blue lines represent standard deviations in each point. These deviations are reduced as we increase the number of samples that are included in the interactions.

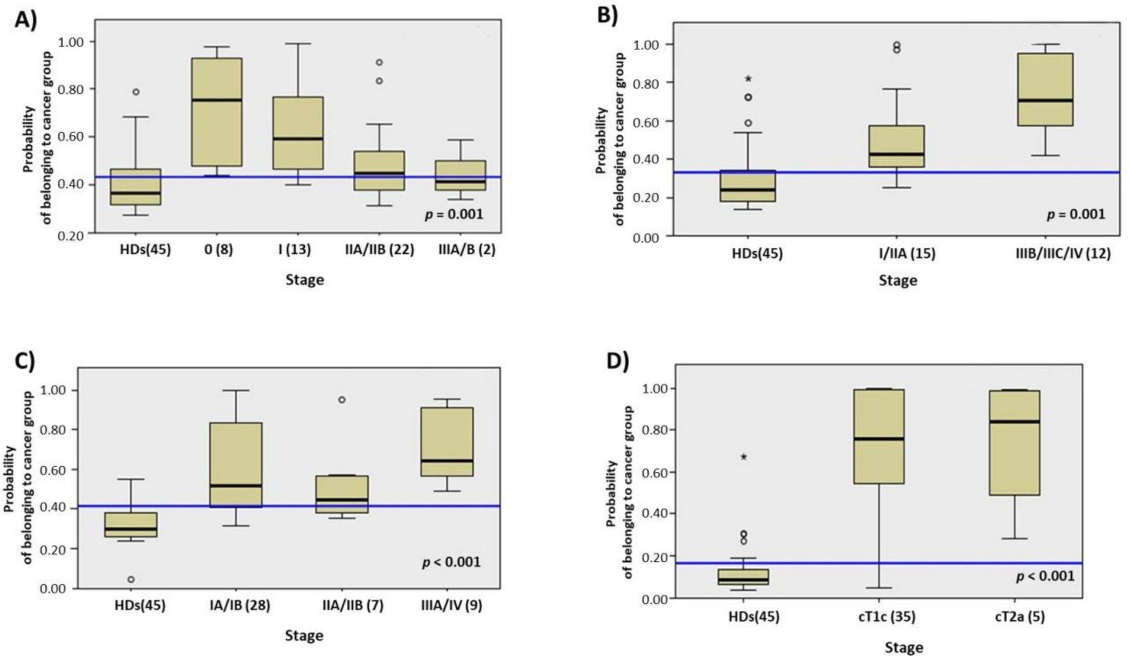

Figure S2. Probabilities of being classified as cancer cases based on the variation of the tumor-selected circulating-miRNA depending on cancer stage. A significant increase in the probability is observed as the tumor stage increases for the four tumor types ($p \leq 0.001$). Test used: non-parametric test for independent samples. The median value for each group is defined by the bold-blue line within the box [A) BC=0.432; B) CRC=0.331; C) LC=0.415; D) PCa=0.165]. Numbers into brackets indicate number of cases in each category.

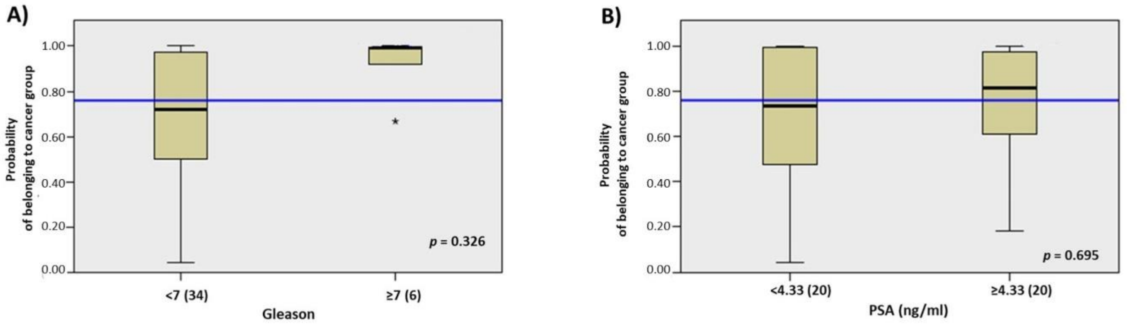

Figure S3. Probabilities of being classified as cancer cases based on the variation of the PCa selected circulating-miRNA depending on GS and PSA values. Although the median values of the probabilities are higher in GS ≥ 7 (**A**) and serum PSA levels above median (**B**), the differences were not statistically significant ($p>0.05$). Test used: non-parametric test for independent samples. The median value for each group is defined by the bold-blue line within the box (GS=0.769; PSA=0.760). Numbers into brackets indicate number of cases in each category.

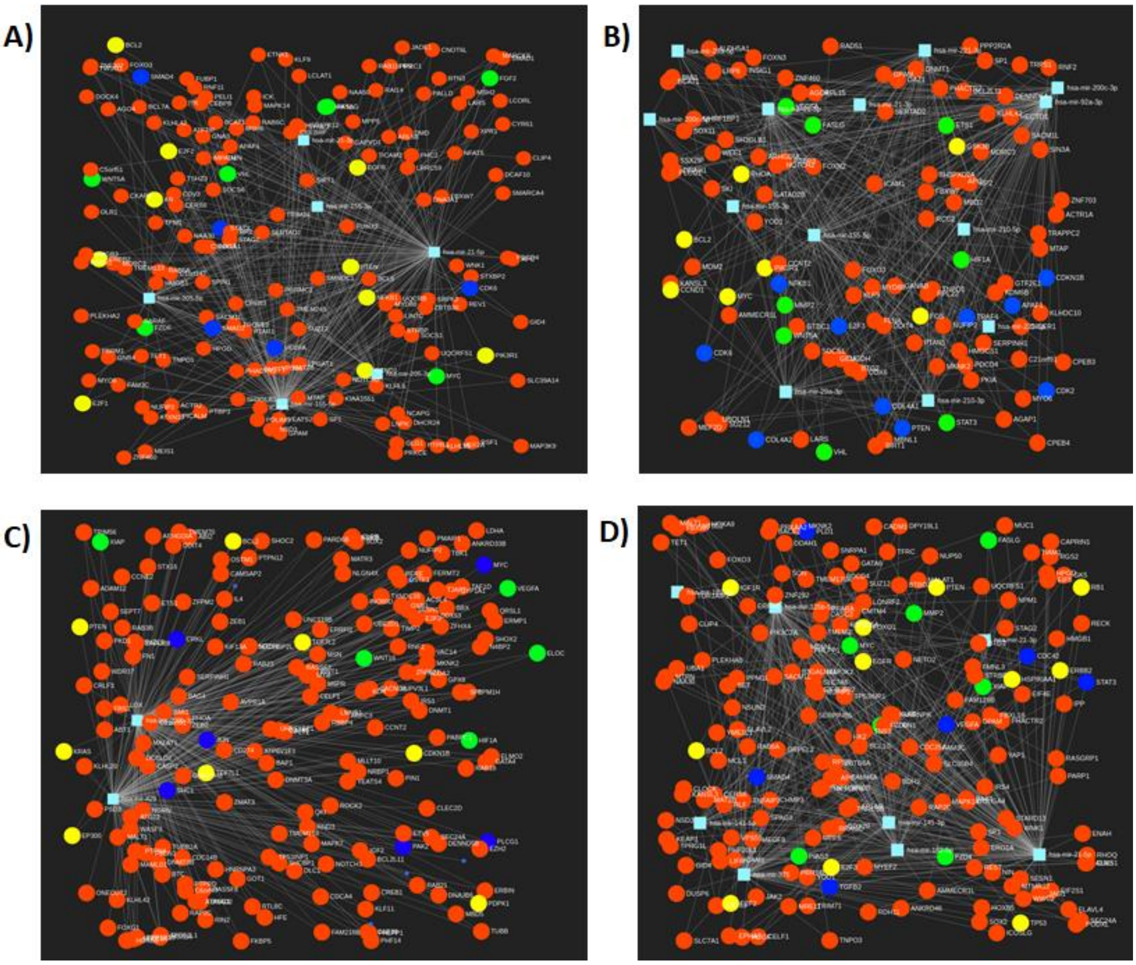

Figure S4. Molecular pathways network showing miRNA interaction with its targets. The three top overrepresented pathways are highlighted in distinct colors. **A**) BC set with pathways in cancer (green), pancreatic cancer (blue) and PCa (yellow); **B**) CRC with pathways in cancer (green), small cell lung cancer (blue) and CRC pathway (yellow). **C**) LC with pathways in cancer (green), ErbB signalling (blue) and PCa (yellow); and **D**) PCa with pathways in cancer (green), pancreatic cancer (blue) and PCa pathway (yellow) highlighted.

References

1. Asaga S, Kuo C, Nguyen T, Terpenning M, Giuliano AE, Hoon DSB. Direct serum assay for microRNA-21 concentrations in early and advanced breast cancer. *Clin Chem*. **2011**;*57*: 84–91.
2. Li S, Yang X, Yang J, Zhen J, Zhang D. Serum microRNA-21 as a potential diagnostic biomarker for breast cancer: a systematic review and meta-analysis. *Clin Exp Med*. **2016**;*16*: 29–35.
3. Toraih EA, Mohammed EA, Farrag S, Ramsis N, Hosny S. Pilot Study of Serum MicroRNA-21 as a Diagnostic and Prognostic Biomarker in Egyptian Breast Cancer Patients. *Mol Diagn Ther*. **2015**;*19*: 179–190.
4. Usmani A, Shoro AA, Memon Z, Hussain M, Rehman R. Diagnostic, prognostic and predictive value of MicroRNA-21 in breast cancer patients, their daughters and healthy individuals. *Am J Cancer Res*. **2015**;*5*: 2484–2490.
5. Yoruker EE, Aydogan F, Gezer U, Saip P, Dalay N. Analysis of circulating microRNAs during adjuvant chemotherapy in patients with luminal A breast cancer. *Mol Clin Oncol*. **2015**;*3*: 954–958.
6. Jiang S, Zhang HW, Lu MH, He XH, Li Y, Gu H, et al. MicroRNA-155 functions as an OncomiR in

breast cancer by targeting the suppressor of cytokine signaling 1 gene. *Cancer Res*. **2010**;*70*: 3119–3127.
7. Sochor M, Basova P, Pesta M, Dusilkova N, Bartos J, Burda P, et al. Oncogenic microRNAs: miR-155, miR-19a, miR-181b, and miR-24 enable monitoring of early breast cancer in serum. *BMC Cancer*. **2014**;*14*: 448.
8. Sun Y, Wang M, Lin G, Sun S, Li X, Qi J, et al. Serum microRNA-155 as a potential biomarker to track disease in breast cancer. *PLoS One*. **2012**;*7*: e47003.
9. Shaker O, Maher M, Nassar Y, Morcos G, Gad Z. Role of microRNAs -29b-2, -155, -197 and -205 as diagnostic biomarkers in serum of breast cancer females. *Gene*. **2015**;*560*: 77–82.
10. Zhang H, Li B, Zhao H, Chang J. The expression and clinical significance of serum miR-205 for breast cancer and its role in detection of human cancers. *Int J Clin Exp Med*. **2015**;*8*: 3034–3043.
11. Yang JS, Li BJ, Lu HW, Chen Y, Lu C, Zhu RX, et al. Serum miR-152, miR-148a, miR-148b, and miR-21 as novel biomarkers in non-small cell lung cancer screening. *Tumour Biol.* **2015**;*36*: 3035–3042.
12. Halvorsen AR, Bjaanæs M, LeBlanc M, Holm AM, Bolstad N, Rubio L, et al. A unique set of 6 circulating microRNAs for early detection of non-small cell lung cancer. *Oncotarget*. **2016**;*7*:37250-37259.
13. Zhang HL, Yang LF, Zhu Y, Yao XD, Zhang SL, Dai B, et al. Serum miRNA-21: elevated levels in patients with metastatic hormone-refractory prostate cancer and potential predictive factor for the efficacy of docetaxel-based chemotherapy. *Prostate*. **2011**;*71*: 326–331.
14. Cheng HH, Mitchell PS, Kroh EM, Dowell AE, Chery L, Siddiqui J, et al. Circulating microRNA profiling identifies a subset of metastatic prostate cancer patients with evidence of cancer-associated hypoxia. *PLoS One*. **2013**;*8*: e69239.
15. Casanova-Salas I, Rubio-Briones J, Calatrava A, Mancarella C, Masiá E, Casanova J, et al. Identification of miR-187 and miR-182 as biomarkers of early diagnosis and prognosis in patients with prostate cancer treated with radical prostatectomy. *J Urol.* **2014**;*192*: 192(1):252-9.
16. Singh PK, Preus L, Hu Q, Yan L, Long MD, Morrison CD, et al. Serum microRNA expression patterns that predict early treatment failure in prostate cancer patients. *Oncotarget*. **2014**;*5*: 824–840.
17. Ogata-Kawata H, Izumiya M, Kurioka D, Honma Y, Yamada Y, Furuta K, et al. Circulating exosomal microRNAs as biomarkers of colon cancer. *PLoS One*. **2014**;*9*: e92921.
18. Zhang G, Zhou H, Xiao H, Liu Z, Tian H, Zhou T. MicroRNA-92a functions as an oncogene in colorectal cancer by targeting PTEN. *Dig Dis Sci*. **2014**;*59*: 98–107.
19. Huang Z, Huang D, Ni S, Peng Z, Sheng W, Du X. Plasma microRNAs are promising novel biomarkers for early detection of colorectal cancer. *Int J Cancer*. **2010**;*127*: 118–126.
20. Tang W, Zhu Y, Gao J, Fu J, Liu C, Liu Y, et al. MicroRNA-29a promotes colorectal cancer metastasis by regulating matrix metalloproteinase 2 and E-cadherin via KLF4. *Br J Cancer*. **2014**;*110*: 450–458.
21. Fasanaro P, D'Alessandra Y, Di Stefano V, Melchionna R, Romani S, Pompilio G, et al. MicroRNA-210 modulates endothelial cell response to hypoxia and inhibits the receptor tyrosine kinase ligand Ephrin-A3. *J Biol Chem*. **2008**;*283*: 15878–15883.
22. Pu XX, Huang GL, Guo HQ, Guo CC, Li H, Ye S, et al. Circulating miR-221 directly amplified from plasma is a potential diagnostic and prognostic marker of colorectal cancer and is correlated with p53 expression. *J Gastroenterol Hepatol*. **2010**;*25*: 1674–1680.
23. Hur K, Toiyama Y, Takahashi M, Balaguer F, Nagasaka T, Koike J, et al. MicroRNA-200c modulates epithelial-to-mesenchymal transition (EMT) in human colorectal cancer metastasis. *Gut*. **2013**;*62*: 1315–1326.
24. Volinia S, Calin GA, Liu CG, Ambs S, Cimmino A, Petrocca F, et al. A microRNA expression signature of human solid tumors defines cancer gene targets. *Proc Natl Acad Sci U S A*. **2006**;*103*: 2257–2261.
